# Mechanistic analysis of MLKL-driven cell survival

**DOI:** 10.1038/s41420-026-03187-8

**Published:** 2026-06-10

**Authors:** Peijia Jiang, Xiaoyang Liu, Mauricio J. Reginato, Kirill V. Rosen

**Affiliations:** 1 Departments of Pediatrics & Biochemistry and Molecular Biology, Dalhousie University, Halifax, NS Canada; 2 AGADA Bioscience, Halifax, NS Canada; 3 Department of Biochemistry and Molecular Biology, Drexel University College of Medicine, Philadelphia, PA USA

**Keywords:** Cell death, Cancer

## Abstract

MLKL pseudokinase is a critical executioner of necroptotic cell death. MLKL drives necroptosis by forming pores in the cell membrane. A growing body of data indicates that, in addition to this well-established role, MLKL can promote cell survival in certain contexts. Moreover, pharmacological or genetic MLKL inhibition was shown to suppress in vivo growth of several tumor types. It was found that MLKL protects cancer cells from various cell death-inducing stimuli by promoting autophagy or preserving the mitochondrial function of the cells. It was proposed that both of these MLKL effects prevent parthanatos, a cell death type mediated by hyperactivation of PARP1 and subsequent PARP1-dependent chromosomal DNA degradation. In addition, MLKL was found to protect tumor cells from the death receptor-induced demise and trigger the secretion of the growth-promoting cytokines by the cells. Notably, MLKL-deficient mice are healthy, while pharmacological MLKL inhibitors are not significantly toxic to mice. Hence, targeting MLKL in vivo to block MLKL-dependent cancer cell survival is feasible. The mechanisms of the pro-survival MLKL effects is the subject of this review.

## Facts

- MLKL is a well-established executioner of necroptotic cell death, but a growing body of data indicates that MLKL can promote cell survival in certain contexts.
- The mechanisms by which MLKL inhibits cell death include MLKL-induced autophagy, MLKL-dependent preservation of the mitochondrial function and MLKL-driven inhibition of parthanatos, a PARP1-mediated form of necrosis.
- MLKL inhibition blocks growth of several tumor types. Moreover, MLKL knockout mice are healthy, and MLKL antagonists are relatively non-toxic in vivo. Thus, targeting MLKL to disrupt MLKL-dependent cancer cell survival represents a viable therapeutic approach.

## Open questions

- To trigger necroptosis, MLKL needs to be phosphorylated by RIPK3 protein kinase [1]. However, the ability of MLKL to protect cells from death, at least in some scenarios, does not require such phosphorylation [2, 3]. In one case, phosphorylation by CaMK2 protein kinase was shown to be necessary for the pro-survival MLKL effect [3]. Which kinases need to phosphorylate MLKL to promote its pro-survival function remains to be explored.
- It also remains to be studied whether the mechanism by which MLKL prevents lipidation of LC3, a major autophagy driver [4], is linked in any way to that via which MLKL promotes autophagosome-to-lysosome fusion.
- MLKL was found to protect cells from death by promoting autophagy and thereby, inhibiting parthanatos, a cell death type characterized by the mitochondrial dysfunction [5]. Whether the ability of MLKL to support the mitochondrial function represents the general consequence of MLKL-driven autophagy is another important question that needs to be answered.
- The ESCRT machinery is regulated by MLKL [6] and at least in some scenarios, MLKL and components of this machinery, e.g., VPS37A [5], play redundant roles in promoting autophagy, while in other cases MLKL drives autophagy upstream of the ESCRT machinery [7]. The precise role of this machinery in the pro-survival effects of MLKL remains to be understood.
- How MLKL-dependent pro-survival mechanisms outlined in this review are related to the ability of MLKL to inhibit death receptor-dependent cell death is another important open question.

## Introduction

Necroptosis is a regulated necrotic cell death occurring in response to bacterial or viral cell infection [1]. Cells dying by necroptosis typically release numerous cytokines, and the major physiological necroptosis function is to trigger immune or inflammatory response as a result of such release [8, 9]. In addition, necroptosis is thought to contribute to heart failure, inflammatory disease and stroke [10–12]. Mixed lineage kinase domain-like (MLKL) pseudokinase is a critical driver of necroptotic cell death [13].

**Fig. 1:**
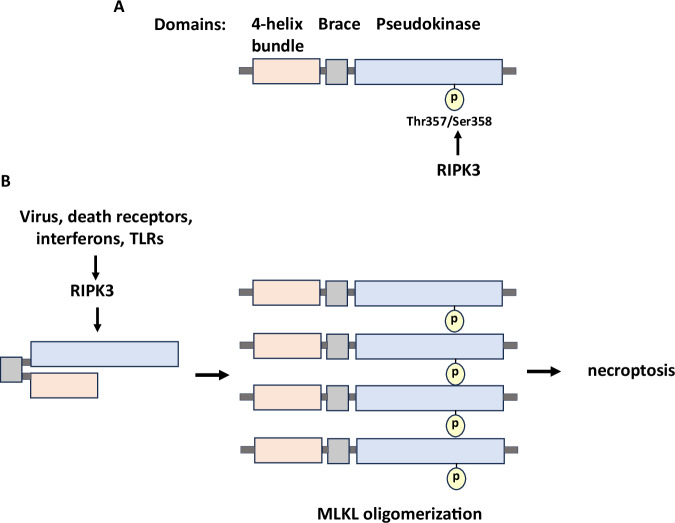
The canonical role of MLKL is to promote necroptosis. **A** Schematic structure of MLKL protein. The four-helix bundle domain, the brace and the pseudokinase domains as well as the sites of phosphorylation of the human MLKL by RIPK3 are shown. **B** Stimuli, such as viral infection, death receptor activation, interferons or Toll-like receptors (TLRs), trigger phosphorylation of MLKL. As a result, MLKL undergoes a conformational change that allows it to oligomerize, form pores in the plasma membrane and thereby cause necroptosis.

The ability of MLKL to cause necroptosis is mediated by its two domains, the N-terminal four-helix bundle and the C-terminal pseudokinase domain that lacks the kinase activity [14] (Fig. 1A). To become active, human MLKL needs to be phosphorylated by the protein kinase RIPK3 at Thr357/Ser358 [15] (Fig. 1A). RIPK3 in turn, can be activated by various stimuli, e.g., viral infection, death receptor activation, interferons or Toll-like receptors (TLRs) [16–18]. In some cases, RIPK3 activation requires its binding to a protein kinase RIPK1 [19]. Activated RIPK3 phosphorylates the pseudokinase domain of MLKL and thereby triggers a conformational change in the domain allowing MLKL to dissociate from RIPK3 [14]. MLKL subsequently forms oligomers (Fig. 1B) and inserts in the plasma membrane due to the interaction between the positively charged residues of the four-helix bundle domain with the negatively charged phosphatidylinositol phosphates present in the membrane. These interactions allow MLKL to form plasma membrane pores and thereby cause necrosis [14] (Fig. 1B). Thus, necroptosis is presently defined as a modality of regulated cell death triggered by perturbations of extracellular or intracellular homeostasis that critically depends on MLKL, RIPK3, and, in some settings, on the kinase activity of RIPK1 [1].

Although MLKL is a well-established executioner of necroptosis [1], mounting evidence indicates that MLKL can protect cells from death in various contexts. Based on these findings, MLKL inhibition has been proposed to represent a novel approach for killing cancer cells [2, 5]. Notably, *MLKL* knockout mice are born with normal Mendelian frequency, do not show any significant phenotype and are healthy as well as fertile [20]. Thus, MLKL inactivation is not toxic to mice, suggesting that MLKL inhibition in tumor cells may kill them without significant systemic toxicity.

MLKL was found to promote cell survival by activating autophagy [3, 5], supporting normal mitochondrial function [3, 21] or increasing cellular degradation of the death receptors [22, 23]. Respective data and potential mechanisms by which MLKL exerts these effects are discussed below.

## Autophagy as a mediator of the pro-survival MLKL effects

**Fig. 2:**
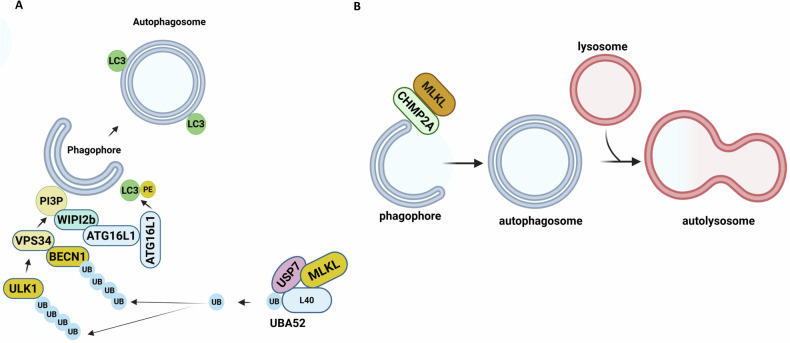
MLKL can promote autophagy. **A** A model of MLKL-dependent LC3 lipidation. MLKL can inhibit lipidation of LC3, an event critical for autophagosome formation. MLKL binds UBA52 composed of ubiquitin and ribosomal protein L40 and thereby recruits ubiquitin-specific peptidase 7 (USP7) to the complex. USP7 further cleaves UBA52 to yield free ubiquitin (UB). This, in turn increases Lys63-linked polyubiquitination of ULK1 as well as BECN1 and enhances stability of these proteins. ULK1 phosphorylates and thereby activates the lipid kinase VPS34. BECN1 needs to bind VPS34 in order for the latter to be active. VPS34 generates pools of PI3Ps which mediate attachment of a protein WIPI2b to the phagophore. WIPI2b further binds a protein ATG16L1 attached to the covalent complex formed by proteins ATG12 and ATG5. The complex drives conjugation of the protein LC3 to a lipid phosphatidylethanolamine (PE). LC3-PE aka LC3II then promotes autophagosome formation. **B** A model of MLKL-dependent LC3 closure of the autophagosome. MLKL can act in complex with or upstream of the component of the ESCRT-III complex CHMP2A to drive autophagosome closure and thereby promote autophagosome-to-lysosome fusion and autophagy.

Macroautophagy, often referred to as ″autophagy″, is the process of degradation of the cellular content in the lysosomes. Such content is brought to the lysosomes by the double membrane-enclosed vesicles called the autophagosomes [1]. To initiate autophagy, ULK1/2 protein kinases phosphorylate and thereby activate the lipid kinase VPS34, which needs to bind a protein BECN1 to be active [4] (Fig. 2A). VPS34 further promotes generation of phosphatidylinositol 3-phosphates (PI3Ps). The latter mediate attachment of a protein WIPI2b to the autophagosome membrane precursor termed the phagophore. WIPI2b subsequently binds a protein ATG16L1 attached to the covalent complex between proteins ATG12 and ATG5 [4]. The complex drives conjugation of a protein LC3 to a lipid phosphatidylethanolamine (PE), and LC3-PE conjugate called LC3II promotes autophagosome formation (Fig. 2A). Once the autophagosome containing material destined for degradation is formed, the autophagosomes fuse with the lysosomes, the autophagosome content is released into the lysosome and is degraded by the lysosomal hydrolyzes [4].

Necrosulfonamide (NSA), a small molecule MLKL inhibitor, binds human MLKL at Cys86, and prevents MLKL oligomerization [13]. NSA treatment or MLKL knockout by CRISPR was found to partially but significantly reduce clonogenic survival of multiple human colorectal cancer cell (CRC) lines [5]. Notably, these effects were substantially enhanced by treatment of the cells with a protein synthesis inhibitor homoharringtonine (HHT), a drug utilized for the management of chronic myeloid leukemia [24]. Furthermore, NSA and HHT cooperated in suppressing the ability of CRC cells to form tumors in immunodeficient mice without being toxic to the animals [5]. In an effort to explain these findings, the authors established that MLKL inhibition and HHT cooperate with each other in suppressing lipidation of the LC3 isoform LC3B, a critical autophagy step, in CRC cells [5]. Furthermore, the cell-permeable peptide derived from the major autophagy driver BECN1 [25] (Fig. 2A) restored cell growth caused by MLKL inhibition combined with HHT treatment indicating that suppression of autophagy caused by this combination treatment plays a causal role in CRC cell growth inhibition [5].

It was further demonstrated that the events outlined above are regulated by the Endosomal Sorting Complexes Required for Transport (ESCRT) machinery [26]. This machinery includes the ESCRT-0, I, -II and III multiprotein complexes that promote the formation of various vesicles, such as intraluminal vesicles, endosomes and autophagosomes [26]. MLKL was found to be able to bind components of the ESCRT-I complex TSG101, MVB12B, VPS28 and VPS37A [27]. Moreover, even in those cases where MLKL drives necroptosis, i.e., plays its canonical role, MLKL was observed to utilize components of the ESCRT-I complex TSG101 and VPS37B as well as those of the ESCRT-III complex CHMP4B, VPS4B and CHMP4A to delay necroptotic cell death by repairing the plasma membrane damaged during necroptosis [6]. It was proposed that MLKL-induced activation of the ESCRT machinery prevents cell demise, until the cells irreversibly commit to death by necroptosis [6].

Notably, it was found that whereas the knockdown of the ESCRT-I component VPS37A does not affect LC3B lipidation in the MLKL-expressing CRC cells, such knockdown dramatically reduces such lipidation in the MLKL-deficient cells [5]. Hence, MLKL and VPS37A seem to play redundant roles in LC3B lipidation. Moreover, autophagy-inhibiting drugs, such as HHT, block this lipidation further when MLKL is inactivated and when VPS37A presence becomes critical for such lipidation [5].

It was found that the combined inactivation of MLKL and HHT treatment increased the population of CRC cells with the hypodiploid sub-G1 DNA content [5], a well-known symptom of programmed cell death [1]. Importantly, the autophagy-inducing BECN1-derived cell-permeable peptide [25] reversed HHT-induced death of MLKL-deficient cells [5]. Thus, MLKL inhibition combined with HHT treatment blocks autophagy in CRC cells, and such autophagy inhibition kills the cells. The mechanism of this death is discussed in the next section.

The ability of MLKL to promote autophagy by driving LC3 lipidation described above is consistent with other studies. For example, it was found that MLKL knockdown by short hairpin (sh)RNAs in mouse neuroblastoma N2a cells, lack of MLKL in the hippocampus derived from MLKL knockout mice or treatment of human embryonic kidney cells HEK293 with NSA inhibits formation of the lipidated LC3 form, thereby signifying reduced autophagy in the cells [28]. Remarkably, in this study, MLKL was shown to promote LC3 lipidation via enhancing the stability of ULK1 and BECN1, two critical drivers of LC3 lipidation (Fig. 2A) [28]. Protein stability is often regulated by ubiquitination. Ubiquitin can be conjugated to the internal lysine residues e.g., Lys11, Lys48 and Lys63 of another ubiquitin molecule [29]. While Lys48-linked polyubiquitin chains typically target proteins for proteasomal degradation, Lys63-linked chains often stabilize the target protein and serve as scaffolds for the recruitment of other proteins [29]. Ubiquitin is synthesized by the ribosomes in a precursor form that is further processed to give rise to active ubiquitin [30]. Four ubiquitin precursors, UBA52, RPS27A/UBA80, UBB and UBC, were identified in mammalian cells [31, 32]. UBA52 represents ubiquitin linked to the ribosomal protein L40 [33], and it was found that MLKL binds UBA52 in the cytoplasm and thereby recruits ubiquitin-specific peptidase 7 (USP7) to the complex. USP7 further cleaves UBA52 to yield free ubiquitin (Fig. 2A). This, in turn increases Lys63-linked polyubiquitination of ULK1 and BECN1, enhances stability of these proteins, and they subsequently trigger LC3 lipidation and thereby promote autophagy [28] (Fig. 2A). Thus, unlike the case with necroptosis where MLKL kills the cells by inserting in the plasma membrane [34], MLKL can also function in the cytoplasm in complex with other proteins, such as UBA52 and USP7 and thereby promote autophagy [28].

In summary, MLKL can promote autophagy by stimulating LC3 lipidation (Fig. 2A). The data outlined above are consistent with the model, whereby MLKL exerts this effect by promoting UBA52 cleavage, release of ubiquitin and subsequent stabilization of ULK1 and BECN1, and the latter proteins then promote conjugation of LC3 with PE and autophagosome formation (Fig. 2A).

Notably, besides driving LC3 lipidation, MLKL can stimulate autophagy by promoting the fusion of autophagosomes with the lysosomes, an autophagy step distinct from LC3 lipidation [3]. For example, it was found that starvation causes MLKL phosphorylation by calcium/calmodulin dependent protein kinase II (CAMK2) in mouse fibroblasts as well as human neuroblastoma and CRC cell lines which allows MLKL to oligomerize and promote autophagy in response to starvation [3]. In this case, the ability of MLKL to drive autophagy was independent of RIPK3. The causal role of MLKL in autophagy was demonstrated by the findings that MLKL gene knockout, MLKL knockdown by RNA interference or pharmacological MLKL inhibition substantially reduced starvation-induced autophagy in the indicated cells [3]. Notably, unlike the case with the studies outlined above, MLKL did not increase LC3 lipidation in this setting but rather enhanced degradation of LC3 and that of the autophagy driver p62 in the lysosomes by accelerating the fusion of the autophagosomes with the lysosomes, another critical autophagy step [3]. Also importantly, starvation caused necrotic death of MLKL-deficient cells much more robustly than that of the MLKL-expressing cells [3]. Thus, MLKL-driven autophagy protects the cells from starvation-induced death.

The ability of MLKL to promote cell growth by accelerating autophagy stages that are distinct from LC3 lipidation is further supported by a study, according to which MLKL allows pulmonary microvascular endothelial cells (PMVECs) to grow in the presence of lipopolysaccharide (LPS) and thereby transiently reduces acute lung injury induced by LPS in mice [7]. It was found that pharmacological MLKL inhibition or small interfering (si)RNA-induced MLKL knockdown inhibits the autophagy flux by suppressing fusion of the autophagosomes with the lysosomes, in LPS-treated PMVECs [7]. LPS treatment was demonstrated to promote incorporation of MLKL in the propagating autophagosomal membrane and accelerate autophagosome closure [7]. This MLKL effect was proposed to be achieved either by MLKL oligomerization or MLKL binding to other yet unidentified proteins. Notably, the ability of MLKL to drive autophagy in this case required the presence of CHMP2A, a component of the ESCRT-III complex, while CHMP2A, when overexpressed in the cells, promoted autophagic flux even in the absence of MLKL activity [7]. Thus, CHMP2A likely mediates autophagy downstream of MLKL in this scenario. Notably, other components of the ESCRT machinery, e.g., VPS37A, were found to mediate autophagosome closure indicating that ESCRT proteins are instrumental in this process [35]. In summary, MLKL can promote autophagosome-to-lysosome fusion, possibly by mediating ESCRT-machinery-dependent autophagosome membrane closure (Fig. 2B).

## Mitochondria as mediators of pro-survival effects of MLKL

**Fig. 3:**
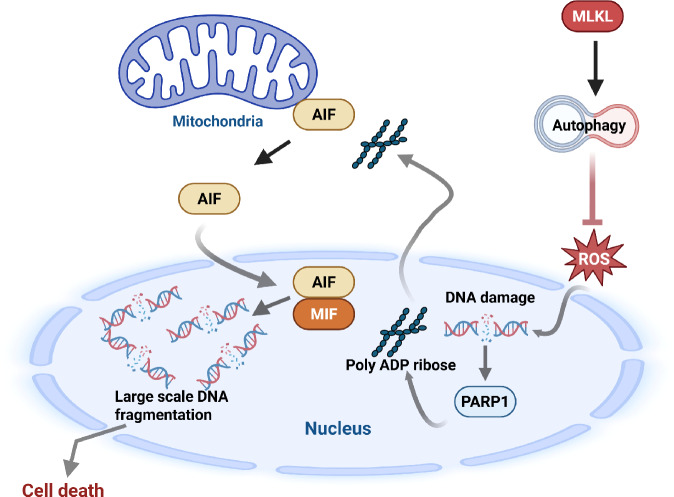
A model of MLKL-dependent parthanatos inhibition. MLKL-induced autophagy downregulates ROS in cells. This inhibits PARP1-dependent PAR formation, PAR-driven release of AIF from the mitochondria, formation of the PAR-MIF complex, subsequent MIF-induced chromosomal DNA fragmentation and the resulting cell death.

It was found that treatment of hepatocellular carcinoma cells (HCC) with palmitic acid (16:0) (PA), the most abundant saturated fatty acid in the human body whose presence is associated with various hepatic diseases, causes reduction in the Mg^2+^ levels in the mitochondria of the MLKL knockout cells much more robustly than in the case of the parental, MLKL-expressing cells [2]. Furthermore, MLKL knockdown triggered mitochondrial dysfunction in the PA-treated cells manifested by the production of reactive oxygen species (ROS) and increased translocation of a cell-death-inducing protein AIF from the mitochondria to the nucleus causing a form of cell death termed parthanatos [2]. Parthanatos is a type of programmed necrosis driven by hyperactivation of poly(ADP-ribose) polymerase 1 (PARP1), which promotes the formation of poly(ADP-ribose) (PAR) [36]. In many cases PAR causes the release of the protein AIF from the mitochondria [36]. AIF then forms a complex with the endonuclease MIF, and the complex causes chromosomal DNA fragmentation and cell death (Fig. 3) [36].

It was demonstrated that PA triggers parthanatos of MLKL knockout HCC cells noticeably more efficiently than that of the MLKL-expressing control cells [2]. Notably, PA-induced parthanatos of MLKL-deficient cells was not blocked by the MLKL mutant that cannot be phosphorylated by RIPK3 indicating that the ability of MLKL to suppress parthanatos is RIPK3-independent in this context [2]. Also notably, MLKL-deficient HCC cells were much less tumorigenic than the respective MLKL-expressing cells when implanted in the liver of the immune-competent mice, and tumorigenicity of the former was restored by treatment with a PARP1 inhibitor olaparib [2]. Furthermore, parthanatos caused by MLKL loss was proposed to block orthotopic tumor growth in mice by inducing anti-tumor immune response characterized by increased infiltration of CD8+ T cells in the tumors. Interestingly, loss of MLKL only weakly reduced tumorigenicity of the cells in the immune-deficient mice. Also remarkably, MLKL-expressing and MLKL-deficient cells displayed similar tumorigenicity when injected in the mice subcutaneously, rather than orthotopically, indicating that lack of MLKL sensitizes the cells to parthanatos triggered by the stressful as-yet unidentified environment specific to the liver [2].

The ability of MLKL to inhibit parthanatos is not unique to HCC cells. As outlined above, the combined inactivation of MLKL and HHT treatment was found to block CRC cell autophagy and thereby kill the cells [5]. This death was prevented by the PARP inhibitor olaparib [37]. Moreover, HHT caused a substantial increase in the cellular PAR species, a parthanatos hallmark [36], in the MLKL-deficient cells [5], and autophagy-inducing BECN1-derived cell-permeable peptide [25] reversed formation of these species in MLKL-deficient cells [5]. These data indicate that MLKL inhibition combined with HHT treatment blocks autophagy in CRC cells, and such autophagy inhibition induces parthanatos. Thus, parthanatos is the emerging mechanism of cell death induced by lack of MLKL [2, 5].

The ability of MLKL to prevent the release of parthanatos-inducing factor AIF from mitochondria is supported by other studies. Acetaminophen is a widely used analgesic and antipyretic drug [38]. Acetaminophen overdose is a frequent cause of acute liver failure [38]. Comparison of the effect of acetaminophen on the wild type and MLKL-deficient mice indicated that MLKL transiently protects mouse liver cells from acetaminophen-induced necrosis and thereby reduces toxicity of acetaminophen to the animals’ liver [38]. It was found that the liver cells derived from the MLKL-deficient cells show a more robust release of AIF from the mitochondria in response to acetaminophen treatment, than the respective control MLKL-expressing cells [38].

The notion that MLKL inhibition can promote mitochondrial dysfunction is further supported by another study. The activity of protein kinase mTOR is stimulated by a GTPase RHEB which, in turn can be inhibited by proteins TSC1 and TSC2 [21]. It was found that TSC2 knockout in mouse embryonic fibroblasts or 621 cells isolated from a human angiomyolipoma sensitizes the cells to necrotic death caused by ROS and mitochondrial dysfunction induced by a combination of oxidative stress inducers auronofin (AF) and buthionine sulfoximine (BSO) [21]. ROS production, mitochondrial dysfunction and cell death were further exacerbated by pharmacological MLKL inhibition or MLKL knockdown by RNA interference [21]. Notably, MLKL inhibition was found to downregulate a metabolite α-ketoglutarate (αKG) in TCS2-deficient cells, and exogenous αKG rescued the cells from NSA-driven death [21]. αKG is a component of the tricarboxylic acid (TCA) cycle [39], and αKG upregulation can elevate the levels of malate, another element of the cycle [39]. Upon exiting the cycle, malate can give rise to NADPH, a well-known oxidative stress inhibitor [39]. Thus, the study again supports the notion that MLKL inhibition can promote mitochondrial dysfunction.

In summary, MLKL can support cell survival by maintaining functional mitochondria and in particular, by preventing mitochondrial dysfunction resulting in the release of AIF from the mitochondria and subsequent parthanatos [2, 38]. Moreover, at least in some contexts, such parthanatos is driven by inhibition of autophagy caused by MLKL loss [5]. How could MLKL prevent AIF release from mitochondria and protect cells from death? Normally, PARP1 repairs DNA damage [36], but excessive damage caused by reactive oxygen species (ROS) hyperactivates PARP1, and PARP1 induces parthanatos [36]. ROS are known to be upregulated by autophagy blockade [40]. Since MLKL inactivation inhibits autophagy [5, 28], it is tempting to speculate that this inhibition induces ROS which in turn trigger parthanatos (Fig. 3).

## Inhibition of the death receptor signaling as a pro-survival mechanism driven by MLKL

It was found that MLKL knockdown by RNA interference strongly sensitizes human cervical, breast, CRC and lung cancer cells to apoptosis caused by the death ligand TRAIL [22]. TRAIL kills cells by activating DR4 and DR5 receptors [1]. To cause apoptosis, TRAIL triggers the assembly of the death-inducing signaling complex (DISC) that includes the TRAIL receptors, the adaptor molecule FADD and initiator caspase 8. When activated by TRAIL at the DISC, caspase-8 promotes apoptosis by triggering the activity of the executioner caspases, e.g., caspase 3 [1]. Notably, the complex containing the receptors and the ligand, including the death receptors and TRAIL, can be internalized by the cells via endocytosis and enters the endosomes which deliver the complex to the lysosomes for degradation [22]. Such degradation inhibits or attenuates TRAIL-dependent pro-apoptotic signals [22].

It was observed that MLKL knockdown in the cell types mentioned above inhibits delivery of the complex containing TRAIL and DR4 to the lysosomes and subsequent degradation of the complex, thereby enhancing TRAIL-dependent pro-apoptotic signals and sensitizing the cells to TRAIL-induced death [22]. In the absence of MLKL, TRAIL promoted the assembly of the DISC more robustly than in the presence of MLKL, presumably because endosome-dependent degradation of the complex components is inhibited in MLKL-deficient cells [22]. Notably, the ability of MLKL to drive degradation of the complex containing TRAIL and DR4 does not seem to require RIPK3 [22].

In further support of the ability of MLKL to inhibit death receptor-induced apoptosis, it was found that MLKL knockdown by RNA interference sensitizes CRC cells to apoptosis induced by chemotherapeutic drug 5-fluorouracil (5-FU) and enhances the ability of the drug to inhibit tumorigenicity of these cells in mice [23]. It was found that 5-FU triggers autocrine production of tumor necrosis factor-α (TNFα) by CRC cells [23]. TNFα activates TNF receptor-I (TNFR-I), another death receptor, that in certain circumstances can trigger capsase-8-dependent apoptosis similarly to TRAIL receptors [1]. TNFR-I turnover is controlled by the endosomes similarly to that of the TRAIL receptors, and MLKL loss was found to prolong TNFR-I retention in the endosomes and delay its degradation further to 5-FU treatment [23]. This, in turn, was proposed to enhance TNF-α/TNFR-I-driven apoptosis of MLKL-deficient cells [23].

**Fig. 4:**
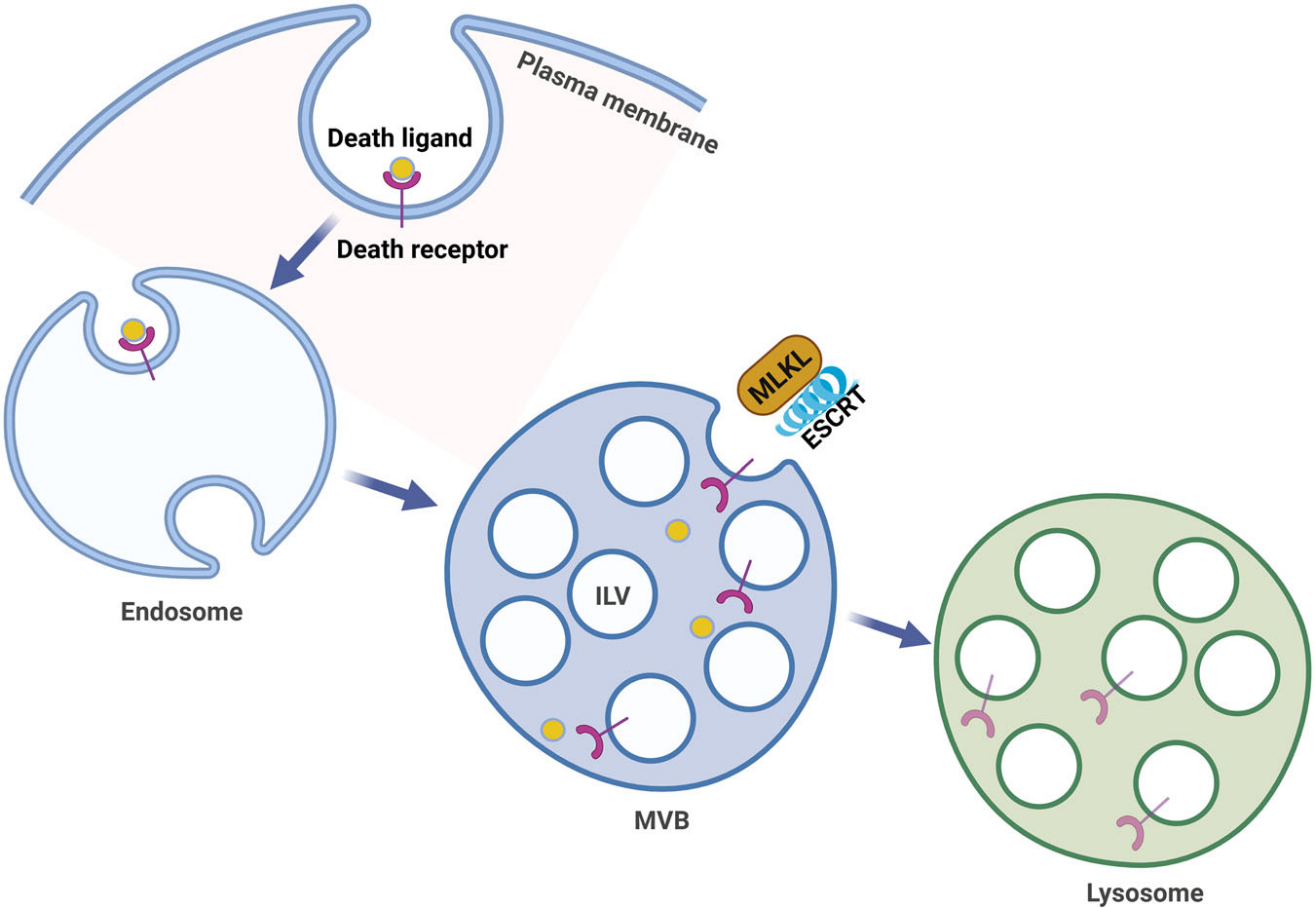
A model of MLKL-dependent inhibition of death receptor signaling. Upon exposure of cells to the death ligands, MLKL cooperates with the ESCRT machinery in sorting of the ligand-receptor complexes to intraluminal vesicles (ILVs). ILVs accumulate within multivesicular bodies (MVBs) which fuse with the lysosomes where the receptor is degraded by the lysosomal hydrolases.

How could MLKL promote endosomal trafficking of the death receptors? A critical step in membrane receptor trafficking is sorting of the receptor present in the endosomes into the intraluminal vesicles (ILVs) which form as a result of the budding of the endosomal membrane [26] (Fig. 4). ILVs then accumulate within multivesicular bodies (MVBs) which fuse with the lysosomes where the receptor is degraded by the lysosomal hydrolases [22]. Sequestration of the cargo and further budding and scission of the endosomal membrane resulting in ILV formation is driven by the ESCRT machinery [22]. It was found that MLKL can bind components of ESCRT-I complex TSG101, MVB12B, VPS28 and VPS37A and promote ILV formation [27]. Thus, it is tempting to speculate that upon exposure of cells to the death ligands, i.e., TRAIL or TNFα, MLKL promotes degradation of the complexes between these ligands and their receptors via ESCRT-dependent sorting of the complexes to ILVs and thereby inhibits such death (Fig. 4). Notably, TRAIL and anti-DR4/5 agonistic antibodies have been explored as anti-cancer drugs but showed low efficacy in the clinic against various cancers [41]. Given the data outlined above, it is conceivable that targeting MLKL could increase the anti-cancer effect of the TRAIL-targeted drugs.

## NFκB-dependent cytokine production as the mechanism of MLKL-driven cell growth

**Fig. 5:**
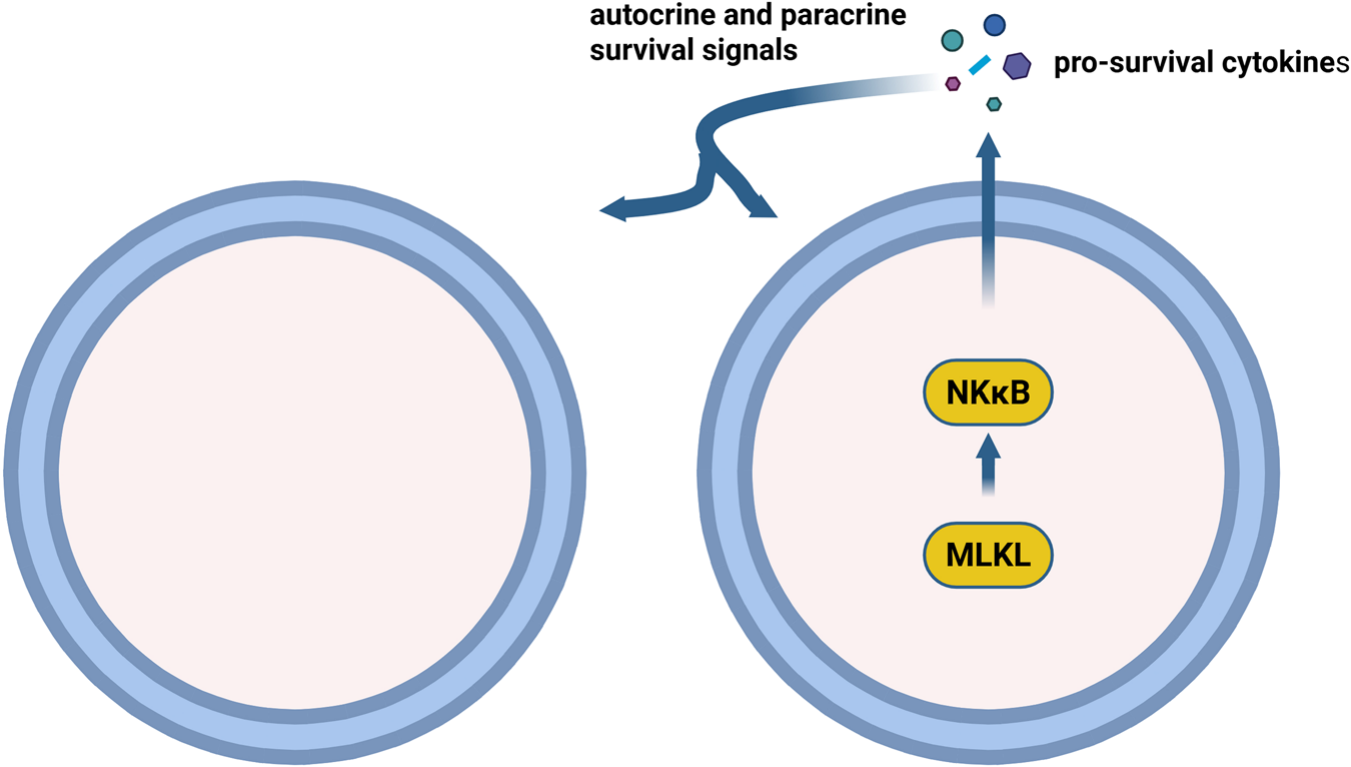
A model of MLKL-dependent stimulation of cancer cell growth via secretion of the cell growth-promoting cytokines. MLKL activates the transcription factor NFkB to trigger secretion of the cell growth-promoting cytokines by the cells. The latter drive cancer cell growth, possibly via both autocrine and paracrine mechanisms.

It was found that while loss of RIPK3 strongly reduced growth of human triple negative breast cancer (TNBC) cells in 2D culture, lack of RIPK1 or MLKL do not significantly affect such growth of these cells. In contrast, knockout of RIPK1, RIPK3 or MLKL by CRISPR substantially reduced the ability of these cells to grow anchorage-independently as colonies is soft agar [42]. Likewise, NSA strongly inhibited anchorage-independent growth of CRC cells. Furthermore, knockout of RIPK1, RIPK3 or MLKL as well as NSA treatment noticeably reduced the ability of TNBC cells to form tumors in immunodeficient mice [42]. In an effort to explain these data, the authors found that knockout of RIPK1, RIPK3 or MLKL genes inhibits production of multiple cytokines, such as IL6, IL8, LCN2, MCP1 and CCL5 by the cells [42]. Furthermore, loss of RIPK1, RIPK3 or MLKL by TNBC cells inactivated the transcription factor NFκB, a known mediator of the production of these cytokines [42]. The authors of the study also observed that the addition of the conditioned medium derived from the wild type TNBC cells to the isogenic variants of the cells lacking RIPK1, RIPK3 or MLKL significantly increased their ability to grow anchorage-independently [42]. A similar effect was achieved if IL6, IL8 or CCL5 were individually added to the cells lacking one of the indicated necroptosis drivers [42]. While the study did not specifically examine whether the indicated treatments affect survival of the cells growing in a 3D manner, the study concludes that MLKL and its activators RIPK1 and RIPK3 promote tumor cell growth and tumorigenicity by mediating the secretion of the pro-survival cytokines by the cells (Fig. 5) [42].

## Unidentified pro-survival mechanisms driven by MLKL

It was found that pharmacological inhibition of MLKL, its ablation by siRNA or acute degradation by the Trim-Away approach (where the protein is degraded by the ubiquitin-proteasome system upon introduction in the cells of the antibody against this protein and the TRIM21 ubiquitin ligase) killed glioblastoma stem-like cells [43]. This death was accompanied by the hallmarks of apoptosis, e.g., increased phosphatidyl serine exposure on the cell surface and activation of caspases-3 and 8. Furthermore, NSA treatment cooperated with a temozolomide, a chemotherapeutic drug used for glioblastoma treatment, in killing the cells. Importantly, NSA treatment substantially suppressed glioblastoma growth in mice and increased survival of glioblastoma-bearing animals [43]. Of note, NSA was not significantly toxic to the mice since the drug did not alter their weight or the blood count and did not affect the weight of their hearts and the kidneys [43]. While the livers of the NSA-treated mice appeared to be slimmer than those of the control animals, the drug did not alter the plasma levels of the liver enzymes typically used to assess the liver function [43]. Notably, MLKL inactivation in the glioblastoma cells also strongly inhibited production of extracellular vesicles (EVs) [43], 80–120 nm lipid layer enclosed bubbles, by the cells [44]. However, addition of EVs secreted by the cells expressing active MLKL to the MLKL-deficient cells did not rescue the latter from death [43]. Hence, inhibition of EV secretion caused by MLKL inactivation does not contribute to death induced by such inactivation in glioblastoma stem-like cells. Thus, the mechanism of the pro-survival MLKL effect remains to be identified in this case.

## Concluding remarks

Multiple studies now indicate that in addition to its well-established role as the executioner of necroptosis, MLKL can protect cells from death, and MLKL inactivation represents a potential approach for killing cancer cells. The mechanisms by which MLKL promotes cell survival have begun to emerge. MLKL can drive autophagy, either by promoting LC3 lipidation [5, 28], a critical autophagy step (Fig. 2A), or by stimulating the fusion of the autophagosomes with the lysosomes [3, 7] (Fig. 2B). Such autophagy, in turn, promotes cells survival. Autophagy blockade inhibits CRC, liver, breast and bladder cancer metastases in mice [45–48], and a lysosome inhibitor hydroxychloroquine [49] is being examined as an autophagy suppressor in numerous clinical trials for various cancers (see clinicaltrials.gov). However, drugs approved for blocking autophagy in cancer are unavailable. Based on the data discussed above, testing whether MLKL inhibition, possibly combined with autophagy antagonists already approved for cancer treatment, e.g., HHT [24], can be used to treat cancer represents a potential direction for the development of novel cancer therapies.

Notably, the ability to regulate both cell survival and autophagy is not unique to MLKL. For example, BCL2, a protein well known to block apoptosis by preventing the release of the mitochondrial pro-apoptotic factors to the cytoplasm [1], can also bind the autophagy driver BECN1 and thereby inhibit autophagy [50]. Likewise, an autophagy driver ATG12 [51] can promote apoptosis via mechanisms similar to those triggered by the pro-apoptotic BCL2 family members [52].

One proposed mechanism of the pro-survival MLKL effect is based on the ability of MLKL to protect the mitochondrial function [2, 21]. Notably, given that the mitochondrial dysfunction in the MLKL-deficient cells triggers parthanatos [2], the same form of death as the one observed in response to inhibition of autophagy caused by MLKL inhibition [5], it is possible the autophagy- and mitochondria-dependent pro-survival MLKL effects are elements of the same mechanism (Fig. 3). It is tempting to speculate MLKL inactivation inhibits autophagy and thereby triggers oxidative stress, a known consequence of autophagy blockade. Conceivably, oxidative stress in turn causes PARP1 hyperactivation and PAR-dependent mitochondrial dysfunction signified by AIF release from the mitochondria and subsequent AIF-dependent chromosomal DNA fragmentation (Fig. 3).

Whether MLKL-dependent autophagy and functional integrity of the mitochondria are related to the ability of MLKL to promote the internalization of the complexes between the death receptors and their ligands (Fig. 4) by cells remains to be established.

The studies outlined above provide the mechanistic basis for exploring MLKL as a target for cancer treatment, where survival of tumor cells could be driven by MLKL. MLKL inhibition suppresses the ability of CRC [5], liver [2], brain [43] and breast [42] cancer cells to form tumors in vivo. Encouragingly, MLKL gene loss or pharmacological MLKL inhibition is not significantly toxic to mice [20], suggesting that MLKL inhibitors could be further studied as novel cancer drugs.

Development of novel MLKL inhibitors is an area of intense research, and numerous compounds are being tested as MLKL antagonists [53, 54]. Perhaps the most widely used MLKL inhibitor is NSA, a drug that covalently binds Cys86 of human MLKL and thereby blocks MLKL oligomerization [13]. The agent inhibits tumorigenicity of CRC cells in immunodeficient mice without reducing their weight, a surrogate indicator of the lack of drug toxicity [5]. Furthermore, NSA does not alter the plasma levels of the liver enzymes in mice and does not affect their blood count or the weight of their hearts and kidneys [43], again, indicating lack of toxicity in vivo. Notably, the mouse MLKL lacks Cys86 critical for NSA binding and thus does not bind NSA [13]. Thus, lack of NSA toxicity due to MLKL inhibition in mice is expected. However, the data outlined above indicate that NSA does not exert any significant toxic off-target effects in the animals supporting potential clinical utility of this MLKL antagonist.

Similar to NSA, several xanthine-based MLKL inhibitors were found to covalently bind Cys 86 of MLKL and inhibit necroptosis of U937 lymphoma and Jurkat leukemia cells [53]. Likewise, a number of sulfonamide compounds were demonstrated to bind MLKL with high affinity and potently inhibit necroptosis of the mouse dermal fibroblasts [53]. Furthermore, the pentacyclic triterpenoid celastrol obtained from a natural product was demonstrated to inhibit MLKL phosphorylation during necroptosis and suppress growth of lung, gastric, CRC, kidney and prostate cancer cells [54]. Testing whether these agents or other recently developed MLKL inhibitors [53, 54] can kill tumor cells and block cancer progression in vivo represents a potential novel direction of the studies aimed at targeting the pro-survival MLKL effects in cancer.
